# Pre-choice midbrain fluctuations affect self-control in food choice: A functional magnetic resonance imaging (fMRI) study

**DOI:** 10.3758/s13415-024-01231-7

**Published:** 2024-10-08

**Authors:** Jakub Skałbania, Łukasz Tanajewski, Marcin Furtak, Todd A. Hare, Marek Wypych

**Affiliations:** 1 Faculty of Psychology, University of Warsaw, Warsaw, Poland; 2 Department of Economics, Kozminski University, Jagiellońska 57, 03-301 Warsaw, Poland; 3 Institute of Cognitive Science, University of Osnabrück, Osnabrück, Germany; 4 Zurich Center for Neuroeconomics, Department of Economics, University of Zurich, Zurich, Switzerland; 5 Laboratory of Brain Imaging, Nencki Institute of Experimental Biology, Polish Academy of Sciences, Warsaw, Poland

**Keywords:** Self-control with food, Brain activity fluctuations, FMRI, Reward system, Value-based decisions, Reference-dependent value

## Abstract

Recent studies have shown that spontaneous pre-stimulus fluctuations in brain activity affect higher-order cognitive processes, including risky decision-making, cognitive flexibility, and aesthetic judgments. However, there is currently no direct evidence to suggest that pre-choice activity influences value-based decisions that require self-control. We examined the impact of fluctuations in pre-choice activity in key regions of the reward system on self-control in food choice. In the functional magnetic resonance imaging (fMRI) scanner, 49 participants made 120 food choices that required self-control in high and low working memory load conditions. The task was designed to ensure that participants were cognitively engaged and not thinking about upcoming choices. We defined self-control success as choosing a food item that was healthier over one that was tastier. The brain regions of interest (ROIs) were the ventral tegmental area (VTA), putamen, nucleus accumbens (NAc), and caudate nucleus. For each participant and condition, we calculated the mean activity in the 3-s interval preceding the presentation of food stimuli in successful and failed self-control trials. These activities were then used as predictors of self-control success in a fixed-effects logistic regression model. The results indicate that increased pre-choice VTA activity was linked to a higher probability of self-control success in a subsequent food-choice task within the low-load condition, but not in the high-load condition. We posit that pre-choice fluctuations in VTA activity change the reference point for immediate (taste) reward evaluation, which may explain our finding. This suggests that the neural context of decisions may be a key factor influencing human behavior.

**Supplementary Information:**

The online version contains supplementary material available at 10.3758/s13415-024-01231-7.

## Introduction

The study of task- and stimulus-evoked brain activities has been a long-standing area of focus in functional magnetic resonance imaging (fMRI) experiments. In these experiments, the brain activities unrelated to the task/stimulus are interpreted as noisy fluctuations and thus ignored or as physiological artifacts and thus eliminated. Conversely, research has demonstrated that the brain exhibits dynamic activity even in the absence of external stimuli or specific mental tasks. Such endogenous activity fluctuations, or resting-state brain activities, reflect the functional organization of the brain, and have often been explored in the context of individual differences (Biswal et al., 1995). However, endogenous fluctuations in brain activity have also been observed in task paradigms (Fox & Raichle, 2007) and have been shown to contribute to the variability in trial-to-trial task-evoked neural activity measured by blood-oxygen-level-dependent (BOLD) imaging (Fox et al., 2006). Given the correlations found between BOLD signals and performance in behavioral or perceptual tasks in fMRI experiments (e.g., Ress & Heeger, 2003), it is possible that the endogenous fluctuations in brain activity contribute to the within-participant variability in performance observed in these experiments.

Indeed, it has been shown that the spontaneous fluctuations in brain activity preceding stimuli presentation, i.e., pre-trial or pre-stimulus brain activity, influences a wide range of behavioral and cognitive processes in humans. These include perception (Boly et al., 2007; Hesselmann et al., 2010a, 2010b; Van Dijk et al., 2008; Wyart & Tallon-Baudry, 2009); cognitive flexibility (Leber et al., 2008); memory encoding (Otten et al., 2006); decision-related processes, for example, perceptual decision-making (Hesselmann et al., 2008, 2010a, 2010b; Hsieh et al., 2012); motor decisions and inhibition (Filevich et al., 2013; Haggard, 2005; Libet, 1985; Libet et al., 1983; Soon et al., 2008); and aesthetic judgment (Colas & Hsieh, 2014). Recent studies have shown that pre-stimulus brain activity influences the complex valuation processes involved in decision-making under risk (Chew et al., 2019; Huang et al., 2014). These results suggest that value-based decision-making, a prominent area of study in neuroeconomics and decision neuroscience (see, e.g., Brosch & Sander, 2013; Hare et al., 2009, 2011; Rangel et al., 2008), may be affected by endogenous fluctuations in brain activity that are unrelated to the decision task. It is therefore possible that decisions may be shaped not only by the attributes of the options selected, but also by the internal brain activity that preceded the choice.

The prospect theory (Kahneman & Tversky, 1979; Tversky & Kahneman, 1991) suggests a mechanism by which pre-choice brain activity may affect decision-making processes. This theory states that the outcomes of a choice are evaluated in relation to a reference point, which enables the decision maker to classify the outcomes as gains or losses and to assess their magnitudes (Kőszegi & Rabin, 2006). A reference point was found to be represented in brain activity during reward evaluation (De Martino et al., 2009). Studies on the reward-prediction error signals in subsequent choices (Pessiglione et al., 2006; Schultz, 1998; Schultz et al., 1997; Thut et al., 1997; Zaghloul et al., 2009) and in tracking the changes in fluctuating reward values in an environment (Wang et al., 2021) suggest that dopaminergic activity may play a role in setting a reference point and in its fluctuations (De Martino et al., 2009; Hamid et al., 2016; Louie & Glimcher, 2012). Chew et al. (2019) showed that a reduction in pre-stimulus endogenous activity in the ventral tegmental area (VTA) was associated with an increased tendency to engage in risk-taking behavior in a series of choices between lotteries. Furthermore, they demonstrated that a lower level of baseline activity in the VTA before the presentation of risky options was predictive of a higher level of phasic activation when gambling decisions were made. The formation of reference-dependent preferences, a topic extensively studied in behavioral economics in relation to risky decision-making (e.g., Kőszegi & Rabin, 2006; O’Donoghue & Sprenger, 2018; Tversky & Kahneman, 1991), could therefore be reflected by the endogenous VTA activity fluctuations prior to decision-making. Specifically, the endogenous activity of the VTA prior to decision-making could establish a “neural context” for reward evaluation during the decision-making process. This was demonstrated by research indicating that more risk-averse decisions were more likely to be made following a higher level of endogenous VTA activity (Chew et al., 2019). This was due to the modulation of task-evoked phasic responses by a pre-existing greater dopaminergic neurons activation, which resulted in a reduction in the reference-dependent valuation of risky options. Contextual valuations have also been observed in other reward-related structures including the nucleus accumbens (NAc), caudate nucleus, globus pallidus, putamen, and frontal brain areas (De Martino et al., 2009; Nieuwenhuis et al., 2005). These regions contribute to decision-making processes through the activation of distinct neural pathways (Hamid et al., 2016; Louie & Glimcher, 2012; Ruff & Fehr, 2014). Consequently, a “neural context” of reward evaluation in each of these regions could influence its region-specific component of value-based decisions and may result in functionally distinct effects of pre-choice activities in these brain regions on subsequent behavior.

The goal of this study was to determine whether pre-choice brain activity (i.e., signals recorded prior to the presentation of choice options to participants) can influence complex value-based decisions that extend beyond those made in risky situations (cf. Chew et al., 2019). In particular, we aimed to ascertain whether reward system activity preceding stimuli presentation is relevant to decisions that require self-control. To investigate self-control in food choice, we used a well-established paradigm to study this problem in an fMRI experiment (e.g., Hare et al., 2009, 2011; Maier et al., 2015; Plassmann et al., 2010; Tanajewski et al., 2023). Our objective was to ascertain whether self-control performance in a binary food choice, defined as choosing a healthier and less tasty food item over a less healthy and tastier one, may be affected by pre-choice activity in the brain reward system.

The reward system is a set of complex and heterogenous structures that work in different ways to influence human behavior (Alonso-Alonso et al., 2015; Arias-Carrión et al., 2010). In addition, different reward system structures are associated with distinct types of neural value representations, and each structure transmits information (directly or indirectly) to the ventromedial prefrontal cortex (VMPFC), which integrates all value signals into a singular value during decision-making processes (Hare et al., 2009, 2011; Levy & Glimcher, 2012; Ruff & Fehr, 2014). Information is transmitted and integrated via various neural pathways (Ruff & Fehr, 2014; Sesack & Grace, 2010). It is therefore impossible to reduce the reward system to a single, functionally cohesive area. We aimed to ascertain how brain activities in various reward system regions involved in self-control may influence subsequent food choices. To this end, we selected four independent regions of interest (ROIs) linked to reward valuation and self-control in previous studies, namely, VTA, putamen, NAc, and caudate nucleus. The VTA is the origin region of dopaminergic neurons in the mesolimbic and mesocortical pathways, which are integral to the processing of rewards. Dopaminergic projections to regions such as the prefrontal cortex (PFC) exert a modulatory influence on PFC activity, thereby supporting the regulation of behavior (Arias-Carrión et al., 2010; Phillips et al., 2008). The VTA is involved in the initial stages of reward prediction, which can be a critical factor even before the commencement of a potentially rewarding task (Schultz, 2007; Wang et al., 2021). Consequently, pre-choice activity in this region may reflect varying levels of dopaminergic activity, thereby establishing the foundation for how upcoming stimuli are evaluated (Chew et al., 2019). The putamen, a component of the dorsal striatum, plays a role in reward processing, response inhibition, and dietary self-control (Dietrich et al., 2016; Haruno & Kawato, 2006; Zandbelt & Vink, 2010). Pre-choice activity fluctuations in the putamen may reflect moment-to-moment changes in the brain’s readiness for inhibition. The NAc, a component of the ventral striatum, is strongly connected with the VTA and PFC and is involved in the mechanisms of reward processing, impulsivity, and self-control (Bartra et al., 2013; Basar et al., 2010; Cartmell et al., 2019; Van der Laan et al., 2011). Furthermore, research has indicated that NAc activity may serve as a pre-stimulus predictor of risky choices (Huang et al., 2014). It may be the case that pre-choice NAc activity reflects baseline reward sensitivity (Berridge & Kringelbach, 2015; Cartmell et al., 2019). The caudate nucleus, another component of the dorsal striatum, plays a key role in reward-related processes, including impulsivity and the anticipation and evaluation of rewards. Caudate nucleus fluctuations may influence how new stimuli are evaluated in relation to current goals and expectations (Babbs et al., 2013; Haruno & Kawato, 2006; Schmidt et al., 2020; Valentin et al., 2007). In light of the aforementioned functional differences between our ROIs, we posited a priori that examining each ROI independently would be advantageous.

The goal of this study was to determine whether neural activity in the selected independent ROIs prior to food choices could predict self-control in these choices. In particular, we aimed to determine whether the selection of a healthier and less tasty food item over a less healthy and tastier one could be predicted. Specifically, we expected pre-choice neural activity to change the reference point for the evaluation of foods attributes, namely, their healthiness and tastiness. This could, in turn, influence the components of value-based food-related decisions that require self-control. We expected the activation of dopaminergic neurons in the reward system prior to decision-making to affect the reference point for the evaluation of immediate rewards, i.e., taste and other immediate hedonic pleasures derived from food. As a result, these food attributes would be valued less in comparison to longer-term rational rewards, such as health. Therefore, we anticipated that the greater pre-choice activity in the selected ROIs would predict a higher probability of successful self-control in food choices. In other words, we hypothesized that the greater brain activity in the VTA, and potentially other key nodes of the reward system as well (putamen, NAc, and caudate nucleus), would be linked with successful self-control.

## Materials and methods

To test our hypotheses, we collected data from an fMRI experiment on self-control in food choice. The widely used paradigm to study self-control in food choice (Hare et al., 2009, 2011) was originally modified to study the neural effects of increased working memory load on self-control (these results will be reported in a separate paper). The hypotheses formulated in this paper and the methods used to check them were developed after the experiment was designed. Accordingly, these hypotheses were not pre-registered or specified prior to data collection. As a result, this study should be treated as an exploratory investigation of the originally pre-planned experiment.

### Participants and procedure

Fifty participants (23 males) aged 18–30 years were recruited for the experiment via online advertisements. All potential participants were screened for exclusion criteria such as contraindications to MRI, a history of mental disorder (including eating disorders), or any neurological illness. Individuals who reported experiencing difficulties with dietary self-control and a general interest in healthy eating were invited to participate in the study. Individuals who adhere to a vegan diet or exclude certain nutrients from their diet for health reasons were not eligible to participate in the study. To ensure consistency in the level of hunger among participants, they were instructed to consume a small meal (e.g., two sandwiches) 2 h prior to the experiment. We obtained written consent from each participant. The procedure was approved by the Kozminski University Ethics Committee (No_2, 2019/2020, 30/06/2020).

First, we collected the subjective healthiness and tastiness ratings of ≤ 414 food items from each participant using a continuous visual scale, which ranged from − 5 (very untasty/unhealthy) through 0 (neutral tastiness/healthiness) to 5 (very tasty/healthy). The order of the tastiness and healthiness ratings was counterbalanced across participants. Based on these ratings, we created 90 pairs of food items, which were unique for each participant: 60 challenging pairs (a healthier and less tasty food item *and* a tastier and less healthy food item) and 30 non-challenging pairs (a healthier and tastier food item *and* a less healthy and less tasty food item). The number of food products evaluated varied between study participants. The rating process continued until the algorithm was able to create the aforementioned 90 food pairs.

Prior to the choice session, participants agreed to select the healthiest available options whenever possible, while also taking into account their own taste preferences. To ensure the realism of the computerized series of food choices, the participants were informed that one randomly selected food item chosen by them would be provided for consumption after the experiment.

**Fig. 1:**
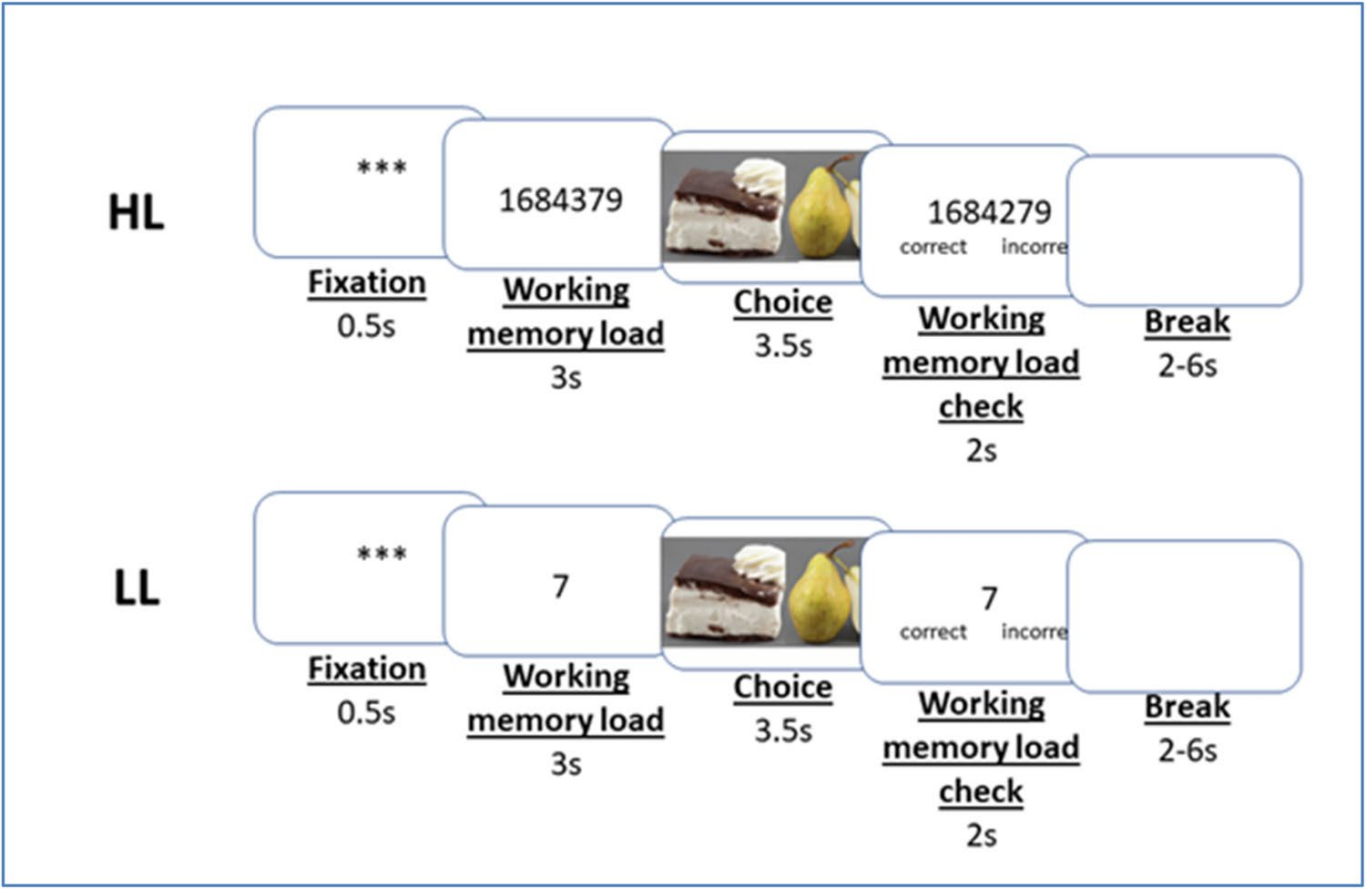
Experimental design. Participants were to memorize a number (working memory load task) and then were presented with a pair of food items, and were to select one of them. At the end of the trial, we verified whether the participants had accurately recalled the number. Each pair of food items was presented twice: once in a high working memory load condition (HL, seven-digit number to be memorized) and once in a low working memory load condition (LL, one-digit number to be memorized)

In the choice session conducted in the fMRI scanner, participants made 90 food choices in two conditions: under low (LL) and high (HL) working memory load. Prior to making each food choice, participants were to memorize a seven-digit number (HL) or a one-digit number (LL) in 3 s. Then they were presented with a pair of food items and had 3.5 s to make a selection. Finally, a number was presented on the screen, and participants were to indicate whether it matched the number they had to memorize. For each correct response, a bonus of 0.01 PLN was added to the participation fee of 120 PLN. See Fig. 1.

In total, 200 trials were conducted with 100 trials per condition: 120 trials with food choices that required self-control (60 challenging pairs presented twice: in HL and LL conditions), for which we calculated pre-choice activity and self-control performance; 60 trials with food choices that did not require self-control (30 non-challenging pairs presented twice: in HL and LL conditions); and 20 trials without any food choices (10 trials in each condition with a blank screen instead of a food choice). The order of both load conditions and the types of pairs (challenging and non-challenging) was fully random and unique for each participant.

In this paper, a working memory task prior to and concurrent with food choices (i.e., memorizing digits before food choice and keeping them in memory during food choice) was an effective method for studying pre-choice brain activity unrelated to food choices. In other words, when participants were to memorize digits before being presented with food items in a choice trial, they were less likely to think about previous or future food choices. A cognitively demanding task irrelevant to food choices eliminated or minimized the risk of brain activities that could influence subsequent choice. For example, research has shown that focusing on the taste or health values of food items before making food choices can influence self-control performance (Hare et al., 2011). In other words, a memory task before food choices forces study participants to cognitively engage in and focus on food-related decision-making in a specific time window only. A similar distractor-based approach to studying the effects of pre-stimulus activity on task performance has been used in previous studies (Colas & Hsieh, 2014; Huang et al., 2014).

### fMRI data acquisition and pre-processing

The MRI data were acquired at the Laboratory of Brain Imaging of the Nencki Institute of Experimental Biology using a 3 T MAGNETOM Trio TIM system (Siemens Medical Solutions) with a whole-head 32-channel coil. Functional data consisting of 973 volumes were acquired using an echo-planar imaging pulse sequence with a multi-band acceleration factor 3. The repetition time (TR) was 1,410 ms, the echo time (TE) was 30.4 ms, the flip angle (FA) was 56°, while the voxel size was 2.5 × 2.5 × 2.5 mm. We obtained the functional data in two runs with the same parameters. Between the two runs, we obtained a structural T1-weighted (T1w) image with a voxel size of 1 × 1 × 1 mm and a TR of 2,530 ms, a TE of 3.32 ms, and a FA of 7°. The entire scanning procedure lasted approximately 55 min.

All imaging data pre-processing was conducted using fMRIPrep 21.0.1 (Esteban et al., 2018a, 2018b; RRID:SCR_016216), which is based on Nipype 1.6.1 (Gorgolewski et al., 2011, 2018; RRID:SCR_002502). We performed the standard fMRIPrep pre-processing with fieldmap correction (based on two (per subject) echo-planar imaging (EPI) references with topup (Andersson et al., 2003); FSL 6.0.5.1:57b01774). The fMRI data were normalized to the MNI space with 2.5 × 2.5 × 2.5 mm voxels. For more details on the fMRIprep pipeline, see fMRIPrep’s documentation at https://fmriprep.org/en/latest/workflows.html.

To prevent contamination of the BOLD signal from structures surrounding our ROIs, we chose not to apply spatial smoothing to our data. It is not advisable to apply smoothing techniques to a priori defined ROI analyses, particularly when the ROI is small and surrounded by heterogeneous tissues (de Hollander et al., 2015).

### Data analysis

A total of 49 out of 50 participants were included in the analysis. One person was excluded from analysis due to the presence of whole-brain artifacts in the fMRI data. The mean age of the participants was 22.1 years (standard deviation (SD) = 2.8). The mean body mass index (BMI) of the participants was 23.0 (SD = 0.4) kg/m^2^. One participant was on a weight loss or maintenance diet and five participants adhered to a vegetarian diet.

#### Behavioral data

Multilevel mixed-effect logistic and linear regressions were used to study the predictors of successful self-control and response time in food choice trials requiring self-control, respectively. The models with a random intercept, random slope, and a random intercept and slope approaches, allowing or not for the correlation between the random slopes and intercepts, were compared using the Akaike Information Criterion (AIC) and Bayesian Information Criterion (BIC) to identify the best model. STATA 18 was used for the analyses.

#### fMRI data

The first-level general linear models (GLMs), estimated using the SPM12 (Wellcome Trust Centre for Neuroimaging, University College, London, UK, http://www.fil.ion.ucl.ac.uk/spm/software/spm12) and Matlab (R2021a) (Mathworks, Inc., Natick, MA, USA), consisted of 14 conditions. The pre-choice events were modeled under HL and LL conditions, before both successful and failed self-control trials (defined for challenging trials only), which resulted in four conditions in the GLM. Also, the events before non-challenging choices in HL and LL conditions were added (two conditions). Challenging and non-challenging food choices, concurrent to HL and LL memory tasks, resulted in four conditions. The memorized numbers were checked against the correct numbers after challenging and non-challenging food choices, in HL and LL conditions, which resulted in four conditions. The ART toolbox was used to add 12 movement regressors (six base motion parameters and their six temporal derivatives) and to regress out motion-affected volumes (frames that exceeded a threshold of 0.5 mm FD or 1.5 standardized DVARS were annotated as motion outliers). Voxels masking threshold for the first-level model was set at 0.25.

The standard GLMs employ the so-called canonical hemodynamic response function (HRF) to model the BOLD signal. However, the HRF convolved with an experimental design results in the modeled signal being shifted in time in relation to the experimental conditions. It is hard to interpret the modeled signal as preceding the choices when such a modeling of the pre-choice BOLD signal is used. Thus, our analyses were conducted using the finite impulse response (FIR) method. The FIR method is not biased towards a particular hemodynamic shape, making it the optimal choice for detecting pre-stimulus signals (Hsieh et al., 2012). In this experiment, each trial was modeled using six finite impulse response predictors (duration = 1.5 s each). The first two FIRs covered a 3-s pre-choice period, FIRs 3–4 covered the choice event, and FIRs 5–6 covered the verification of the memorized digits. Thus, the pre-choice activity was defined as the mean of the betas across 2 time points in the period of 3 s preceding the presentation of food options.

We were interested in the pre-choice activity prior to challenging trials in the pre-defined ROIs. The VTA ROI was defined based on the probabilistic atlas of human subcortical nuclei (http://neurovault.org/collections/3145/) (Pauli et al., 2018). The anatomical masks for the left and right NAc, left and right putamen, and left and right caudate nucleus were specified according to the IBASPM71 atlas implemented in the WFU PickAtlas (Maldjian et al., 2003) toolbox, version 2.4.

We were interested in identifying any differences in the pre-choice activity before self-control success and failure trials (main effect), regardless of working memory task condition (another factor). Therefore, contrast estimate values for ROIs were extracted using a MarsBaR toolbox (Brett et al., 2002) based on the subject-level SPM models from four conditions preceding challenging choices: pre-choice activity prior to self-control success (SC) in HL, pre-choice activity prior to self-control failure (NOSC) in HL, pre-choice activity prior to self-control success (SC) in LL, and pre-choice activity prior to self-control failure (NOSC) in LL. The values from the bilateral ROIs were averaged across the right and left hemispheres because they were highly correlated. Extracted values were normalized to preserve the between-condition differences within each participant, but to remove possible differences in activity levels between participants. Specifically, we standardized each value with the mean and SD across four conditions for each ROI and for each participant. The standardized contrast estimate values were then used to perform fixed-effects logistic regression, with the aim of examining the associations between pre-choice brain activities in our ROIs and the probability of self-control success in the subsequent food choice task. The model was constructed with a binary outcome variable indicating whether or not self-control was exercised (defined as choosing a healthier food item over a tastier one). The regression predictors were the working memory load condition (HL or LL), pre-choice brain activities in each of our ROIs, and the interaction between memory load and activity in each ROI. The data were analyzed using STATA18.

Note that the logistic regression analysis tests for marginal effects of pre-choice activity in each ROI on subsequent self-control behavior. In other words, the multiple regression analysis quantifies the additional effect of each ROI after accounting for the variance explained by the other three ROIs. For a complementary analysis of each ROI independently using repeated-measures ANOVA, see Online Supplementary Materials (OSM).

## Results

### Behavioral results

The mean percentages of successful self-control (± SD) were 67.2% ± 12.2 and 66.8% ± 12.0 in the HL and LL conditions, respectively, and there was no significant difference between the memory load conditions (p = 0.729). See Table S1 in OSM.

The response times were longer in failed self-control trials compared to successful self-control when controlling for the load level (p < 0.001), with longer response times in the low- compared to the high-load condition (p = 0.004); the interaction between load level and self-control performance was not significantly associated with response time (p = 0.183). See Table S2 in OSM.

We did not observe strong carry-over effects of the previous trial type or behavior on current decisions at the behavioral level. We tested whether self-control performance in a given trial was affected by the memory load condition and choice type (challenging or non-challenging) in a previous trial. Our analysis revealed no significant effects of memory load or choice type (see Table S3 in OSM). Next, we examined whether self-control performance (in a given challenging trial) was affected by self-control performance in a previous challenging trial, or by a healthier choice in a previous non-challenging trial. Again, we did not find any significant effects (see Table S4 in OSM).

### fMRI results

**Fig. 2:**
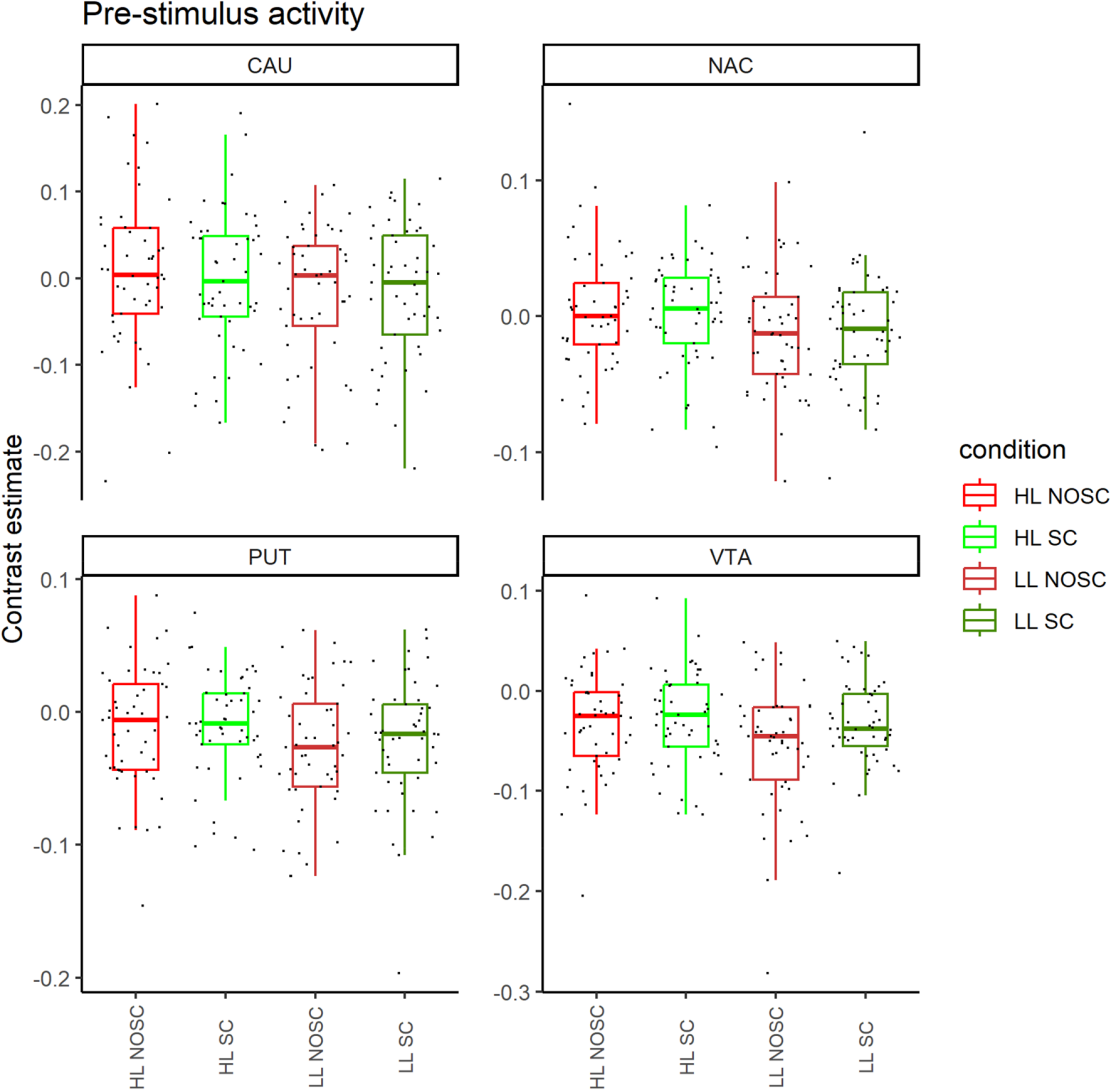
The contrast estimates values of pre-choice activity in all regions of interest (ROIs) (averaged over hemispheres). High-load condition with failed self-control trials (HL NOSC); high-load condition with successful self-control trials (HL SC); low-load condition with failed self-control trials (LL NOSC); low-load condition with successful self-control trials (LL SC). *CAU* caudate nucleus, *NAL* nucleus accumbens, *PUT* putamen, *VTA* ventral tegmental area. The lower and upper hinges of the boxplots correspond to the first and third quartiles. The upper whisker corresponds to 1.5 * IQR (inter-quartile range)

Pre-choice activity in VTA was associated with greater self-control success. Logistic regression analyses indicated a main effect of pre-choice VTA activity on self-control success (z-score = 3.42, p = 0.001) as well as an interaction between memory load and pre-choice VTA activity (z-score = −3.03, p = 0.002; see Table 1 and Fig. 2). Given the details of our regression specification, these results indicate that higher pre-choice VTA activity was associated with self-control success in the low load condition, but that this association was not significant in the high memory load trials. We confirmed this interpretation in post hoc regressions using only low or high memory load trials (see Table 2).

**Table 1:** Results from a logistic regression with self-control success in a subsequent food choice task as a dependent variable

	Coefficient	95% CI	z-score	p-value
VTA	1.134	0.483, 1.785	3.42	0.001
NAc	− 0.163	− 0.722, 0.395	− 0.57	0.566
Caudate nucleus	− 0.805	− 1.600, − 0.009	− 1.98	0.047
Putamen	0.576	− 0.085, 1.237	1.71	0.088
HL	− 0.225	− 0.782, 0.332	− 0.79	0.429
VTA*HL	− 1.325	− 2.181, − 0.469	− 3.03	0.002
NAc*HL	0.153	− 0.743, 1.049	0.34	0.737
Caudate nucleus*HL	0.952	− 0.114, 2.018	1.75	0.080
Putamen*HL	− 0.574	− 1.510, 0.360	− 1.20	0.228
Model statistics		χ2 = 5.86; p-value of χ2 test = 0.32; AIC = 176.171; BIC = 205.674		

*HL* high memory load condition, *LL* low memory load condition

**Table 2:** Results from two separate logistic regressions testing low (LL) and high (HL) memory load trials independently. In these regressions, self-control success in a subsequent food choice task was the dependent variable. Pre-choice activities in NAc, caudate nucleus, and putamen were included as the regressors in both analyses. No significant associations between these activities and self-control success were found when testing only LL or only HL trials. Thus, we omit the results for the sake of brevity

	Coefficient	95% CI	z-score	p-value
HL VTA	− 0.008	− 0.403, 0.387	− 0.04	0.969
LL VTA	1.027	0.351, 1.703	2.98	0.003
HL model statistics	χ2 = 0.12; p-value of χ2 test = 0.998; AIC = 75.804; BIC = 86.144			
LL model statistics	χ2 = 15.26; p-value of χ2 test = 0.004; AIC = 60.671; BIC = 71.011

In addition to the VTA results, higher pre-choice activity in the caudate nucleus was associated with a lower probability of self-control success in a subsequent food choice task (z-score: −1.98, p = 0.047) in the full regression model (see Table 1). However, these caudate results did not reach traditional significance levels in the separate regressions testing high and low memory load trials independently (p = 0.056) (see Table S16 in OSM). More importantly, no significant relationship between pre-choice caudate activity and self-control was found in complementary analyses testing each ROI independently from the other three (see OSM for these repeated-measures ANOVA results). This suggests that the apparent relationship between pre-choice caudate activity and self-control success may be biased by simultaneously accounting for activity of the VTA, NAc, and putamen. We note that the association between pre-choice VTA and self-control success is consistent across all analysis methods. Thus, in contrast to these consistent VTA effects, we suggest caution in interpreting the potential effects of pre-choice activity in the caudate.

We did not find any significant associations between pre-choice activities in other ROIs (see Table 1 and OSM).

We include the results of a complementary analysis of each ROI independently (repeated-measures ANOVA) in the OSM. These results indicate higher pre-choice VTA activity before self-control success compared to self-control failure. No significant associations were found for any other ROIs.

## Discussion

The goal of the present study was to determine whether pre-choice brain activity in the key reward system areas can influence decisions that require self-control. We conducted an experiment with a series of binary food choices and analyzed the VTA, NAc, putamen, and caudate nucleus activities prior to the choices that required self-control. Specifically, we examined the ability to choose long-term goals (health) over immediate rewards (taste). We hypothesized that elevated pre-choice activity in the VTA would increase the likelihood of success in the subsequent self-control task. Our analysis showed that the interaction between pre-choice VTA activity and high memory load was associated with a lower probability of self-control success in the subsequent food choice task. Post hoc tests revealed that pre-choice activity was associated with self-control success in the low-load condition, but not in the high-load condition. Specifically, greater VTA activity was linked to successful self-control in the subsequent food-choice task in the low-load condition. It is noteworthy that the results for the caudate nucleus exhibited a marginally significant effect in a direction contrary to our initial expectations. In other words, greater pre-choice activity in the caudate nucleus was linked to a higher probability of self-control failure in a subsequent food-choice task. However, the pre-choice activities in the putamen and NAc were not found to be significantly associated with self-control performance in subsequent food choice trials. Our findings suggest that increased VTA activity prior to the presentation of food options may influence food-related decisions toward improved self-control. Our findings reinforce the notion that the within-subject variability observed in decision-making may arise from fluctuations in brain activity caused by both endogenous and exogenous factors (cf. Chew et al., 2019; Colas & Hsieh, 2014; Hamid et al., 2016; Huang et al., 2014).

The results of the previous study and their proposed interpretation (Chew et al., 2019) offer a potential explanation for one of our findings: a higher pre-choice activity in the VTA before successful self-control in the low-load condition. Chew et al. (2019) showed that a reduction in pre-stimulus VTA activity increased the probability of choosing risky options. This effect could be attributed to the influence of the pre-stimulus BOLD response on the magnitude of the phasic BOLD response when a decision under risk was made (during their choice task trials). Specifically, a lower level of endogenous VTA background activity (pre-stimulus) was found to be predictive of a greater BOLD response to the presentation of choice options. In light of the notion that the value of a reward is computed in the brain in relation to an ever-changing reference point (De Martino et al., 2009; Kahneman & Tversky, 1979; Kőszegi & Rabin, 2006; O’Donoghue & Sprenger, 2018; Tversky & Kahneman, 1991), and in reference to the studies on the role of the brain reward system in the processes of reward valuation (Pessiglione et al., 2006; Schultz, 1998; Schultz et al., 1997; Thut et al., 1997; Wang et al., 2021; Zaghloul et al., 2009), the authors proposed that the VTA dopaminergic activity before the presentation of stimuli may act as a contextual background to which the subsequent stimulus-related rewards are compared when computing the reward values in the brain (Chew et al., 2019). We postulate that a comparable mechanism may be involved in value-based decisions requiring self-control. The changes in VTA activity, caused by both endogenous and exogenous factors, shift a temporary reference point to which immediate rewards (taste) are compared, while a reference point for the evaluation of longer-term benefits (health) is unchanged. This “neural context” of the immediate reward valuation modulates the magnitude of the neural response to the immediately rewarding stimuli presentations (in this case, high-resolution colorful food images) when food choices are made. This, in turn, affects self-control performance. Specifically, increased activation of dopaminergic neurons in the VTA prior to food choices could affect the reference point for the evaluation of taste rewards, reducing the value placed on the tastiness of foods compared to their health attributes. This would explain why we observed increased pre-choice activity in the VTA before successful compared to failed self-control trials. In this way, fluctuations in VTA activity may be one of the mechanisms underlying inconsistencies in food-related decisions that require self-control, as well as in any value-based decisions that require self-control (i.e., those involving immediate rewards other than the taste of foods).

It is also possible that a reference-dependent evaluation of rewards is driven by the neurophysiological mechanisms described by the classical model of homeostatic dopamine system regulation (Grace, 1991, 1995, 2000). Specifically, a tonic dopamine release modulates a phasic response to stimuli through the constant activation of postsynaptic receptors and autoreceptors. Consequently, an increase in tonic activity results in a reduction in the magnitude of phasic responses to stimuli (Grace, 1991, 1995, 2000). If the pre-stimulus endogenous VTA activity partially reflects tonic activity, these neurophysiological mechanisms could contribute to explaining Chew’s (2019) findings. Furthermore, if our expectation is correct that a reference point for the evaluation of immediate rewards (taste) rather than long-term benefits (health) is encoded in the pre-choice VTA activity, our findings on self-control in food choice may also be explained. Indeed, Chew et al. (2019) showed that participants responded faster in high compared to low pre-stimulus activity conditions. This may be attributed to a higher tonic dopamine level associated with a higher pre-stimulus activity (Hamid et al., 2016; Niv et al., 2007). It was not possible to replicate these results within the constraints of our design. However, we found that our participants exhibited a faster response time in food choices during successful compared to failed self-control trials. This suggests that response times in trials that were preceded by higher compared to lower VTA activity were shorter. It is important to note that the fMRI design used to study pre-choice activity may have inherent methodological limitations, which could potentially impact the interpretation of the observed activity as purely tonic activity (see *Limitations and future directions*” below).

The originally planned experiment was designed to study the effect of increased working memory load on self-control. The hypotheses presented in this paper were formulated subsequent to the completion of the original experiment’s design, which included the incorporation of a working memory task. We speculated that this exploratory study could benefit from a dual-task design (in which the working memory task was concurrent with food choices), which might prevent participants from engaging in task-irrelevant mind wandering. However, the results indicated that the pre-choice VTA activity was more pronounced in the low memory load trials, suggesting that the design may not be optimal for the intended purpose. It may be advisable for future experiments to use only low memory loads or other mild distractors that do not require significant cognitive resources.

On the other hand, the present results raise the intriguing question of why an increased memory load diminished the effect of pre-choice activity in the VTA on self-control success in subsequent choice. Several potential explanations for this finding exist. First, it is possible that making self-control decisions in a more mentally taxing environment (as represented by the high-load condition in our experiment) could diminish the effect of computing the value of the reward relative to a reference point. In other words, under conditions of increased memory load, pre-choice activity in the reward system may become irrelevant to the subsequent choice outcome as a result of the changes in neural activity that are directly linked to effort or self-control. Second, the introduction of a more demanding concurrent task (such as in the high-load condition of our experiment) could result in greater variability in the neural activity patterns (i.e., noise), thereby rendering the subtle differences between activity preceding successful and failed self-control trials more challenging to detect. Third, it is possible that participants were not fully distracted from the series of food choice tasks when performing the less demanding task. Consequently, the pre-choice activity in the low-load condition may have reflected the participants’ expectations about the next trial or their thoughts about the preceding trials. Such expectations may potentially exert an influence on subsequent decisions.

Expectation components in pre-choice activities may potentially enhance the effect of VTA activity on subsequent self-control. For example, greater satisfaction derived from previous instances of successful self-control, which is likely linked with heightened VTA activity, could potentially enhance the probability of successful self-control in a subsequent choice.^1^ Nevertheless, we postulate that these explanations are implausible, given that the outcomes of previous choices are not related to subsequent choice outcomes (see Table S4 in OSM).

Additionally, our findings indicate that elevated levels of pre-choice caudate nucleus activity may be associated with reduced subsequent self-control. This is contrary to our initial hypothesis that reward system activity is associated with enhanced self-control. However, the preliminary findings for the caudate nucleus were considerably less robust than the effects observed in the pre-choice VTA activity. This association only achieved the level of traditional statistical significance (p = 0.047) in the multiple regression analysis. Furthermore, the strength and direction of the relationship between caudate activity and self-control exhibited inconsistency across analyses (as evidenced by the repeated-measures ANOVA results – see OSM). It can thus be concluded that this result is not sufficiently convincing and should be interpreted with caution, given that it is most likely driven by simultaneously accounting for the activity of the other ROIs in the regression. If future research replicates this effect, it may be attributed to the involvement of the caudate nucleus in goal-directed behaviors. The literature indicates that caudate activation is linked to several key behaviors and processes, including delayed gratification (Benningfield et al., 2014), goal-directed approaches (Tanaka et al., 2008), perceived control over outcomes (Tricomi et al., 2004), and selective inhibition (Schmidt et al., 2020). We therefore propose that pre-choice caudate activity may reflect tonic activity that modulates phasic activity during decision-making. It is plausible that increased pre-choice caudate activity may result in a reduction in neural responses during food choice tasks, which could ultimately lead to a decline in self-control. In conclusion, we propose that the pre-choice caudate nucleus may potentially exert an influence on the reference point for the valuation of long-term goals. Future research is required to investigate this hypothesis.

The pre-choice activity in the VTA, rather than in striatal nuclei, was found to be linked with the outcomes of subsequent choices. In light of these findings, we propose that the VTA may serve as the source of fluctuations in the brain reward system, potentially influenced by both endogenous and exogenous factors, which contribute to the variability observed in human decision-making. This conclusion is in line with previous findings indicating the complex and heterogenous roles of the brain structures associated with the reward system in reward valuation processes (Alonso-Alonso et al., 2015; Arias-Carrión et al., 2010).

The finding that heightened pre-choice activity in the VTA, but not in other regions of the reward system, was associated with subsequent self-control success in our study can be attributed to a number of potential explanations. For example, Kroemer et al., (2014, 2016) showed that while higher cue-induced activations in the NAc, vmPFC, and amygdala were predictive of greater effort in subsequent motor-response tasks, higher activities in the VTA and SN (substantia nigra) were predictive of lower effort in these tasks. This suggests that the striatum and VTA may have distinct roles with respect to reward computation and anticipation. The NAc encodes information regarding the level of effort required to obtain the reward, whereas the VTA/SN encodes the value of the reward *minus* the effort level needed to gain it. In light of these findings, an alternative interpretation of the results may be proposed. If pre-choice activity in the VTA is more closely associated with immediate reward (taste), then lower activity prior to the presentation of the food item can be linked to a higher level of effort exerted to obtain the immediate reward. Consequently, a reduction in pre-choice activity in the VTA would result in failed self-control in the subsequent food choice task. An alternative hypothesis is that BOLD responses in the VTA may be influenced by norepinephrine inputs from the locus coeruleus, a region involved in self-control, which projects to the VTA but not to the striatum (Grueschow et al., 2022; Malenka et al., 2009). Although our present data do not allow us to distinguish between these hypotheses, they represent promising avenues for future investigation.

### Limitations and future directions

One limitation of this study is related to the properties of hemodynamic response in the fMRI analyses. To prevent the observed BOLD signals from being contaminated by the preceding trials, it is recommended that a break of approximately 18 s should be taken between trials (Lindquist et al., 2009). In this study, the intertrial intervals (ITI) lasted 2–6 s, with a fixation interval of 0.5 s, resulting in a considerably shorter time interval than 18 s between our trials (i.e., between digits check in a previous task and memorizing digits in a current task). It is therefore possible that in a pre-choice activity in a current trial we may still be observing signals from previous trials, which could complicate the interpretation of our results. In other words, it would not be possible to ascertain whether the observed effect of pre-choice VTA activity on subsequent self-control should be interpreted as resulting from endogenous or exogenous fluctuations in reward system activity. It should be noted that, even if this is the case, both the order of food choice conditions (challenging or non-challenging) and the order of memory load conditions were randomly assigned and unique for each participant. Additionally, the ITI duration was random, which should *average out* any potential artifacts from the previous trial signals at both the individual and the group levels. To test this hypothesis at the behavioral level, we conducted an analysis to ascertain whether a specific type of previous trial (high vs. low load, challenge vs. no challenge) or a participant’s decision in a previous trial (self-control success vs. failure in challenging trials or a healthier choice in non-challenging trials) affected a participant’s performance in the subsequent self-control task. No such effects were identified. In light of these findings, the results of our behavioral analyses lend support to the aforementioned expectation of *averaging out* the unwanted artifacts in our data. This provides a rationale for the pre-choice activity observed in this study to be at least partially endogenous. In other words, it can be concluded that this brain activity was spontaneous and unrelated to the task. It is also important to take into account the drawbacks associated with using an ITI of 18 s in our study. Even if it were to rule out the possible effect of exogenous factors on the observed BOLD activity, it would introduce the possibility that a participant’s thoughts were unrelated to our experimental tasks or – in the case of some participants – engaged in in-depth deliberations about food choices they were making within the context of the experiment. Both scenarios would present challenges to our interpretation, as it would be difficult to control for the brain activities associated with these thoughts. In conclusion, a longer undisturbed break may also increase the risk that the observed brain activity was not the result of spontaneous brain activity, but rather reflected a coherent line of thought. One potential solution would be to use a distractor during a longer break (to prevent participants from engaging in non-task-related thoughts). However, given the required number of trials for powerful analyses, this approach would render the experiment impractically lengthy and exhausting for participants.

It is noteworthy that the preceding pre-stimulus studies were conducted using both extended, uninterrupted ITIs (Chew et al., 2019) and a shorter break (with and without a distractor task) (Colas & Hsieh, 2014; Hsieh et al., 2012; Huang et al., 2014). In view of the potential advantages and disadvantages inherent in the use of both methods, it may prove beneficial to employ both approaches to gain a deeper understanding of the role played by endogenous fluctuations in brain activity with regard to the variability observed in human behavior.

Methodological compromises and limitations are inherent to fMRI and are similarly present in many other types of experiments. The limitations of our present design may give rise to reasonable questions regarding our interpretation of the pre-choice activity measured as tonic activity. It must be acknowledged that we have not fully disentangled tonic from phasic influences on VTA pre-choice activity. Accordingly, future studies are required to address these methodological constraints and further explore the proposed explanations for our findings. Specifically, in addition to studies with longer ITIs, experiments employing manipulations that affect neural activity in the brain reward system would be beneficial in exploring the tonic versus phasic VTA activities and their influence on subsequent behavior. For example, studies employing neurofeedback that would focus on VTA activity could prove to be a promising approach to developing this idea. Previous studies have shown that individuals may exert volitional control over VTA activity (Hellrung et al., 2022; Kirschner et al., 2018; MacInnes et al., 2016; Sulzer et al., 2013). Therefore, a future investigation into the impact of pre-choice VTA activity on self-control in food choice may benefit from instructing participants to volitionally decrease or increase VTA activity until specific activity thresholds are reached, after which food items could be presented and choices could be made. Such paradigms, which employ neurofeedback or those that use real-time fMRI without neurofeedback manipulation (as previously discussed by Chew et al., 2019), may prove to be more sensitive tools for detecting the impact of pre-choice activity on subsequent choices. That is, in such experiments, choices are made only after activity reaches low or high thresholds. Consequently, larger differences in pre-choice activities may affect decisions more than the between-trial differences in pre-choice activity studied using our paradigm. However, it is important to acknowledge the limitations of such designs. In addition to the previously discussed potential for mind wandering during longer ITIs, which introduces noise to the analyses, the use of neurofeedback or real-time fMRI is limited to studies focused on a single ROI at a time. Consequently, studies that aim to examine multiple ROIs, as in our exploratory study, may be impractically lengthy. This is due to the necessity of repeating each choice session for each ROI under investigation. Nevertheless, we posit that the findings from our exploratory, multiple-ROI-focused study can serve as a foundation for future research. A real-time fMRI (rt-fMRI) or neurofeedback design could prove a valuable follow-up to our study.

The series of computerized food choices has been widely used in the fMRI studies on self-control (e.g., Hare et al., 2009, 2011; Maier et al., 2015; Plassmann et al., 2010). Food choices that require self-control serve as illustrative examples for the study of value-based decisions, as the healthiness and tastiness of foods well describe the attributes of the options under consideration. Nevertheless, future studies focused on other decisions, either on self-control in a non-food domain or on decisions unrelated to self-control, would be required to generalize our conclusions and to strengthen the interpretations of our findings. Specifically, further investigation is required into the complex processes of value-based decision-making, using experiments on choices with well-defined and measurable choice attributes. This will enable a deeper understanding of the ideas proposed to explain the results observed. For example, such studies would facilitate a more comprehensive understanding of the role of pre-choice reward system activity in the formation of bias in decision-making, in a mechanism of setting a reference point for reward evaluation. Furthermore, they would enable further investigation of the specific role of the VTA in the endogenous reward system fluctuations relevant to the variability or inconsistency in human decision-making.

## Conclusions

In line with the existing literature and our hypothesis, we have shown that the pre-choice brain activity can influence subsequent behavior. The present study focused on food-related decisions that necessitate self-control, and the expected pattern was observed in VTA activity prior to choice trials. Specifically, a higher level of activity before the presentation of the food items was linked to a greater probability of self-control success in the subsequent food-choice task, indicating the potential for stronger self-control. Our findings lend support to the notion that value-based decision-making processes may be partly affected by internal brain activity in the reward system observed prior to decision-making, which is independent of the attributes of the chosen options. This challenges the traditional (classical) view of utility-driven decisions. However, in light of previous studies in behavioral economics and decision neuroscience, we have applied the concept of a reference-dependent reward valuation to explain our results. In other words, we proposed that dopaminergic activity prior to stimulus presentation could serve as a contextual background or neural context, providing a reference point for the valuation of the subsequent stimulus-related rewards. Future studies are needed to further explore this idea. Finally, our findings suggest that fluctuations in the brain reward system may originate from the VTA, potentially influenced by both endogenous and exogenous factors. These fluctuations may contribute to variability and inconsistencies in human decision-making.

## Supplementary Information

Below is the link to the electronic supplementary material.

Supplementary file1 (DOCX 124 kb)

## Data Availability

Data are available upon request.

## Footnotes

1 Participants could also experience regret from failed self-control in a previous choice, which may be associated with decreased VTA activity, and which may decrease the chances for successful self-control in a subsequent choice.

## Code availability

Codes are available upon request.
