# Supplementary material for: Pre-choice midbrain fluctuations affect self-control in food choice: A functional magnetic resonance imaging (fMRI) study
Source: Cogn Affect Behav Neurosci. 2024 Oct 8;25(2):387–401. doi: 10.3758/s13415-024-01231-7 (PMC11906498; doi:10.3758/s13415-024-01231-7)
Supplement: Supplementary file 1 — Supplementary file1 (DOCX 124 kb) [file 13415_2024_1231_MOESM1_ESM.docx]

**Behavioral results**

Multilevel mixed-effect logistic and linear regressions were used to study the predictors of successful self-control (Tables S1 and S3-S4) and response time (Table S2), respectively. The models with a random intercept, random slope, and a random intercept and slope approaches, allowing or not for the correlation between the random slopes and intercepts, were compared using the Akaike Information Criterion (AIC) and the Bayesian Information Criterion (BIC) to select the best model. The analyses were conducted using STATA 18.

|  | **Coefficient** | **95% CI** | **z-score** | **p-value** |
| --- | --- | --- | --- | --- |
| High load | 0.020 | −0.092, 0.132 | 0.35 | 0.729 |
| Constant | 0.738 | 0.575, 0.902 | 8.84 | < 0.001 |
| Model statistics | χ2 = 0.12; p-value of χ2 test = 0.729; AIC = 7220.628 ; BIC = 7240.656 | | | |

Table S1. The effect of high compared to low load on self-control success (N = 49, 60 choice trials).

Notes: A random intercept approach was applied.

|  | **Coefficient** | **95% CI** | **z-score** | **p-value** |
| --- | --- | --- | --- | --- |
| High load | −0.027 | −0.045, −0.029 | −2.91 | 0.004 |
| Successful self-control | −0.113 | −0.142, −0.084 | −7.69 | < 0.001 |
| Constant | 7.190 | 7.137, 7.242 | 7.19 | < 0.001 |
| Model statistics | χ2 = 67.73; p-value of χ2 test < 0.0001; AIC = 1789.524; BIC = 1836.257 | | | |

Table S2. The effect of load and successful self-control on response time (N = 49, 60 choice trials).

Notes: Given the absence of an effect of load on self-control, we included both load and self-control as variables of interest in the model. A random intercept and slope approach, which did not allow for the correlation between the random slopes and intercepts, was applied. No interaction between load and self-control was found (p-value = 0.183; AIC = 1791.751 and BIC = 1851.836 for the model with interaction). The natural logarithm of response time was used, as this resulted in a lower AIC and BIC value compared to the models using response time.

In our study, the intertrial intervals (ITI) were shorter than the time required for the hemodynamic response to return to baseline. Given that the order of trial conditions (high load or low load and self-control challenging and non-challenging) was randomly assigned and unique for each participant, we reasoned that any activity artifact coming from the previous trials would be averaged out. To verify this expectation at the behavioral level, we conducted analyses to ascertain whether a specific type of previous trial (high vs. low load, challenge vs. no challenge) or a participant’s decision in a previous trial (self-control success vs. failure in challenging trials or a healthier choice in non-challenging trials) affected a participant’s performance in the subsequent self-control task. See Tables S3 and S4.

|  | **Coefficient** | **95% CI** | **z-score** | **p-value** |
| --- | --- | --- | --- | --- |
| High load in a previous trial | −0.017 | −0.130, 0.095 | −0.30 | 0.763 |
| Challenge in a previous trial | 0.026 | −0.089, 0.141 | 0.44 | 0.657 |
| Constant | 0.747 | ~~4~~0.568, 0.926 | 8.19 | < 0.001 |
| Model statistics | χ2 = 0.21; p-value of χ2 test = 0.866; AIC = 7175.81; BIC = 7202.502 | | | |

Table S3. The effect of load and a type of choice (challenging vs. non-challenging) in a previous trial on self-control success (N = 49, 60 choice trials).

Notes: A random intercept approach was applied. No interaction between load and self-control was found (p-value = 0.57; AIC = 7177.495 and BIC = 7210.851 for the model with interaction).

| **Challenge in a previous trial** | | | | |
| --- | --- | --- | --- | --- |
|  | **Coefficient** | **95% CI** | **z-score** | **p-value** |
| Successful self-control in the previous trial | 0.078 | −0.080, 0.235 | 0.97 | 0.333 |
| Constant | 0.715 | 0.520, 0.909 | 7.20 | < 0.001 |
| Model statistics | χ2 = 0.94; p-value of χ2 test = 0.333; AIC = 4279.361; BIC = 4297.815 | | | |
| **No challenge in a previous trial** | | | | |
|  | **Coefficient** | **95% CI** | **z-score** | **p-value** |
| Healthier choice in the previous trial | −0.042 | −0.329, 0.245 | −0.28 | 0.~~4~~76 |
| Constant | 0.762 | 0.470, 1.054 | 5.12 | < 0.001 |
| Model statistics | χ2 = 0.08; p-value of χ2 test = 0.776; AIC= 2224.059; BIC = 2240.495 | | | |

Table S4. The effect of successful self-control (challenging trials) and of a healthier choice (non-challenging trials) in a previous trial on self-control success (N = 49).

Notes: Since there was no effect of load on self-control (see Table S1) or on healthier choice (in non-challenging trials), we did not include load in the model. A random intercept approach was applied for both models.

**2. fMRI results**

Our main analysis was focused on the effect of pre-choice activity on self-control performance. For the sake of transparency, we also present a complementary method of analysis that we conducted prior to modifying our analytical approach to fixed effects logistic regression. We conducted a repeated measures ANOVA. Our objective was to ascertain whether there was a difference in the pre-choice activity preceding trials in which self-control success and failure were achieved (main effect), regardless of working memory task condition (another factor). Therefore, contrast estimate values for ROIs were extracted using a MarsBaR toolbox (Brett et al., 2002) based on the subject-level SPM models from four conditions preceding challenging choices: pre-choice activity prior to self-control success (SC) in HL, pre-choice activity prior to self-control failure (NOSC) in HL, pre-choice activity prior to self-control success (SC) in LL, and pre-choice activity prior to self-control failure (NOSC) in LL. The extracted values were subjected to a repeated measures ANOVA in JASP (version 0.17.1) with two factors: Self-control (SC or NOSC) and Load (HL or LL). The ANOVA analyses revealed similar results. However, after correcting for multiple ROIs, the main effect of pre-choice activity on self-control would no longer reach statistical significance. In light of the aforementioned considerations, we present the uncorrected p-values.

The pre-choice activity in the VTA (but not in the other ROIs) differed between self-control success and failure trials (p = 0.045) and was higher in the high load compared to the low load condition (p = 0.028). No significant interaction between self-control success and load was found (p = 0.113). See Table S5-S7.

The pre-choice activity in the left NAc did not significantly differ between successful and failed self-control (p = 0.289), nor between the high load and low load conditions (p = 0.098). No significant interaction between self-control success and load was found (p = 0.721). See Table S8.

The pre-choice activity in the right NAc did not significantly differ between successful and failed self-control (p = 0.506) and was higher in the high load compared to the low load condition (p = 0.004). No significant interaction between self-control success and load was found (p = 0.691). See Table S9 and S10.

The pre-choice activity in the right caudate nucleus did not significantly differ between successful and failed self-control (p = 0.54), nor between the high load and low load conditions (p = 0.045). No significant interaction between self-control success and load was found (p = 0.318). See Table S11.

The pre-choice activity in the caudate nucleus did not significantly differ between successful and failed self-control (p = 0.956), nor between the high load and low load conditions (p = 0.059). No significant interaction between self-control success and load was found (p = 0.681). See Table S12.

The pre-choice activity in the right putamen did not differ between successful and failed self-control (p = 0.695), nor between the high load and low load conditions (p = 0.026). No interaction between self-control success and load was found (p = 0.687). See Table S13.

The pre-choice activity in the left putamen did not significantly differ between successful and failed self-control (p = 0.360) and was higher in the high load compared to the low load condition (p < 0.001). No significant interaction between self-control success and load was found (p = 0.628). See Table S14 and S15.

| **VTA activity: within subjects effects** | | | | | | | |
| --- | --- | --- | --- | --- | --- | --- | --- |
| **Cases** | **Sum of Squares** | | **df** | **Mean Square** | **F** | **p-value** | **η²_p_** |
| Self-control | 0.009 | | 1 | 0.009 | 4.258 | 0.045 | 0.081 |
| Residuals | 0.097 | | 48 | 0.002 |  |  |  |
| Load | 0.009 | | 1 | 0.009 | 5.119 | 0.028 | 0.096 |
| Residuals | 0.088 | | 48 | 0.002 |  |  |  |
| Self-control x Load | 0.004 | | 1 | 0.004 | 2.333 | 0.133 | 0.046 |
| Residuals | | 0.082 | 48 | 0.002 |  |  |  |
| *Note.* Type III Sum of Squares | | | | | | | |

Table S5. Mean VTA activity: repeated measures ANOVA with two factors (self-control performance and memory load).

Notes: Load: high load (HL) and low load (LL) working memory conditions. Self-control: successful self-control (SC) and failed self-control trials (NOSC).

| **Post hoc comparison of the effect of self-control in the VTA** | | | | | |
| --- | --- | --- | --- | --- | --- |
|  | **Mean Difference** | **SE** | **t** | **p-value** | **Cohen’s d** |
| SC - NOSC | 0.013 | 0.006 | 2.063 | 0.045 | 0.258 |

Table S6. The mean difference in VTA activity prior to successful and failed self-control, averaged over the levels of memory load (post hoc comparison).
Note: SE - standard error. Self-control: successful self-control (SC) and failed self-control trials (NOSC).

| **Post hoc comparison of the effect of load in VTA** | | | | | |
| --- | --- | --- | --- | --- | --- |
|  | **Mean Difference** | **SE** | **t** | **p-value** | **Cohen’s d** |
| HL - LL | 0.014 | 0.006 | 2.263 | 0.028 | 0.269 |

Table S7. The mean difference in VTA activity between the high and low memory load conditions, averaged over the levels of self-control (post hoc comparison).
Note: SE - standard error. Load: high load (HL) and low load (LL) working memory conditions.

| **Within Subjects Effects – Left NAc** | | | | | | | |
| --- | --- | --- | --- | --- | --- | --- | --- |
| **Cases** | **Sum of Squares** | | **df** | **Mean Square** | **F** | **p** | **η²_p_** |
| Self-control | 0.002 | | 1 | 0.002 | 1.150 | 0.289 | 0.023 |
| Residuals | 0.066 | | 48 | 0.001 |  |  |  |
| Load | 0.004 | | 1 | 0.004 | 2.851 | 0.098 | 0.056 |
| Residuals | 0.059 | | 48 | 0.001 |  |  |  |
| Self-control x Load | 1.456×10^-4^ | | 1 | 1.456×10^-4^ | 0.129 | 0.721 | 0.003 |
| Residuals | | 0.054 | 48 | 0.001 |  |  |  |
| *Note.* Type III Sum of Squares | | | | | | | |

Table S8. Mean left NAc activity: repeated measures ANOVA with two factors (self-control performance and memory load).

Note: Load: high load (HL) and low load (LL) working memory conditions. Self-control: successful self-control (SC) and failed self-control trials (NOSC).

| **Within Subjects Effects – Right NAc** | | | | | | | |
| --- | --- | --- | --- | --- | --- | --- | --- |
| **Cases** | **Sum of Squares** | | **df** | **Mean Square** | **F** | **p** | **η²_p_** |
| Self-control | 4.405×10^-4^ | | 1 | 4.405×10^-4^ | 0.450 | 0.506 | 0.009 |
| Residuals | 0.047 | | 48 | 9.794×10^-4^ |  |  |  |
| Load | 0.017 | | 1 | 0.017 | 9.408 | 0.004 | 0.164 |
| Residuals | 0.086 | | 48 | 0.002 |  |  |  |
| Self-control x Load | 2.065×10^-4^ | | 1 | 2.065×10^-4^ | 0.160 | 0.691 | 0.003 |
| Residuals | | 0.062 | 48 | 0.001 |  |  |  |
| *Note.* Type III Sum of Squares | | | | | | | |

Table S9. Mean right NAc activity: repeated measures ANOVA with two factors (self-control performance and memory load).

Notes: Load: high load (HL) and low load (LL) working memory conditions. Self-control: successful self-control (SC) and failed self-control trials (NOSC).

| **Post hoc comparison of the effect of load in right NAc** | | | | |  |
| --- | --- | --- | --- | --- | --- |
|  | **Mean Difference** | **SE** | **t** | **p-value** | **Cohen’s d** |
| HL - LL | 0.019 | 0.006 | 3.067 | 0.004 | 0.406 |

Table 10. The mean difference in right NAc activity between the high and low memory load conditions, averaged over the levels of self-control (post hoc comparison).
Note: SE - standard error. Load: high load (HL) and low load (LL) working memory conditions.

| **Within Subjects Effects – Right caudate nucleus** | | | | | | | |
| --- | --- | --- | --- | --- | --- | --- | --- |
| **Cases** | **Sum of Squares** | | **df** | **Mean Square** | **F** | **p** | **η²_p_** |
| Self-control | 0.001 | | 1 | 0.001 | 0.381 | 0.540 | 0.008 |
| Residuals | 0.178 | | 48 | 0.004 |  |  |  |
| Load | 0.022 | | 1 | 0.022 | 4.219 | 0.045 | 0.081 |
| Residuals | 0.249 | | 48 | 0.005 |  |  |  |
| Self-control x Load | 0.005 | | 1 | 0.005 | 1.018 | 0.318 | 0.021 |
| Residuals | | 0.227 | 48 | 0.005 |  |  |  |
| *Note.* Type III Sum of Squares | | | | | | | |

Table S11. Mean right caudate nucleus activity: repeated measures ANOVA with two factors (self-control performance and memory load).

Note: Load: high load (HL) and low load (LL) working memory conditions. Self-control: successful self-control (SC) and failed self-control trials (NOSC).

| **Within Subjects Effects – Left caudate nucleus** | | | | | | | |
| --- | --- | --- | --- | --- | --- | --- | --- |
| **Cases** | **Sum of Squares** | | **df** | **Mean Square** | **F** | **p** | **η²_p_** |
| Self-control | 1.379×10^-5^ | | 1 | 1.379×10^-5^ | 0.003 | 0.956 | 6.485×10^-5^ |
| Residuals | 0.213 | | 48 | 0.004 |  |  |  |
| Load | 0.018 | | 1 | 0.018 | 3.736 | 0.059 | 0.072 |
| Residuals | 0.230 | | 48 | 0.005 |  |  |  |
| Self-control x Load | 7.964×10^-4^ | | 1 | 7.964×10^-4^ | 0.171 | 0.681 | 0.004 |
| Residuals | | 0.223 | 48 | 0.005 |  |  |  |
| *Note.* Type III Sum of Squares | | | | | | |  |

Table S12. Mean left caudate nucleus activity: repeated measures ANOVA with two factors (self-control performance and memory load).

Note: Load: high load (HL) and low load (LL) working memory conditions. Self-control: successful self-control (SC) and failed self-control trials (NOSC).

| **Within Subjects Effects – Right putamen** | | | | | | | |
| --- | --- | --- | --- | --- | --- | --- | --- |
| **Cases** | **Sum of Squares** | | **df** | **Mean Square** | **F** | **p** | **η²_p_** |
| Self-control | 2.233×10^-4^ | | 1 | 2.233×10^-4^ | 0.156 | 0.695 | 0.003 |
| Residuals | 0.069 | | 48 | 0.001 |  |  |  |
| Load | 0.005 | | 1 | 0.005 | 5.307 | 0.026 | 0.100 |
| Residuals | 0.044 | | 48 | 9.134×10^-4^ |  |  |  |
| Self-control x Load | 1.847×10^-4^ | | 1 | 1.847×10^-4^ | 0.165 | 0.687 | 0.003 |
| Residuals | | 0.054 | 48 | 0.001 |  |  |  |
| *Note.* Type III Sum of Squares | | | | | | | |

Table S13. Mean right putamen activity: repeated measures ANOVA with two factors (self-control performance and memory load).

Note: Load: high load (HL) and low load (LL) working memory conditions. Self-control: successful self-control (SC) and failed self-control trials (NOSC).

| **Within Subjects Effects – Left putamen** | | | | | | | |
| --- | --- | --- | --- | --- | --- | --- | --- |
| **Cases** | **Sum of Squares** | | **df** | **Mean Square** | **F** | **p** | **η²_p_** |
| Self-control | 9.929×10^-4^ | | 1 | 9.929×10^-4^ | 0.853 | 0.360 | 0.017 |
| Residuals | 0.056 | | 48 | 0.001 |  |  |  |
| Load | 0.013 | | 1 | 0.013 | 13.972 | < 0.001 | 0.225 |
| Residuals | 0.044 | | 48 | 9.151×10^-4^ |  |  |  |
| Self-control x Load | 3.053×10^-4^ | | 1 | 3.053×10^-4^ | 0.238 | 0.628 | 0.005 |
| Residuals | | 0.062 | 48 | 0.001 |  |  |  |
| *Note.* Type III Sum of Squares | | | | | | | |

Table S14. Mean left putamen activity: repeated measures ANOVA with two factors (self-control performance and memory load).

Notes: Load: high load (HL) and low load (LL) working memory conditions. Self-control: successful self-control (SC) and failed self-control trials (NOSC).

| **Post hoc comparison of the effect of load in the left putamen** | | | | | |
| --- | --- | --- | --- | --- | --- |
|  | **Mean Difference** | **SE** | **t** | **p-value** | **Cohen’s d** |
| HL - LL | 0.016 | 0.004 | 3.738 | < 0.001 | 0.217 |

Table S15. The mean difference in left putamen activity between the high and low memory load conditions, averaged over the levels of self-control (post hoc comparison).
Note: SE - standard error. Load: high load (HL) and low load (LL) working memory conditions.


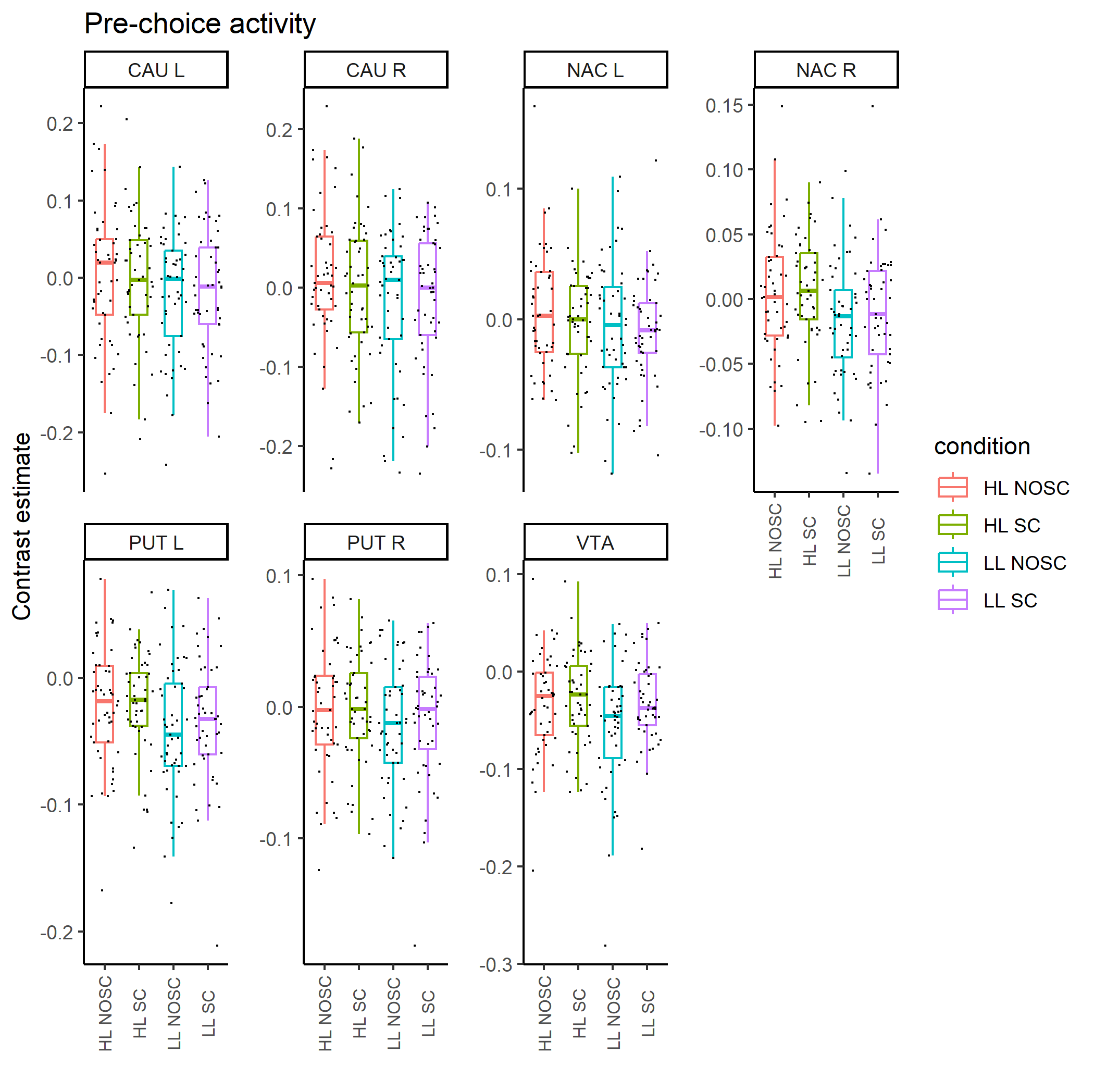


Figure S1. Contrast estimate values of pre-choice activity in all ROIs. High load condition with failed self-control trials (HL NOSC); high load condition with successful self-control trials (HL SC); low load condition with failed self-control trials (LL NOSC); low load condition with successful self-control trials (LL SC). CAU – caudate nucleus; NAL – nucleus accumbens; PUT – putamen; VTA – ventral tegmental area. The lower and upper hinges of the boxplots correspond to the first and third quartiles, respectively. The upper whisker corresponds to 1.5 * IQR (inter-quartile range).

|  | **Coefficient** | **95% CI** | **z-score** | **p-value** |
| --- | --- | --- | --- | --- |
| HL Caudate | −0.001 | −0.480, 0.476 | −0.01 | 0.994 |
| LL Caudate | −0.860 | −1.741, 0.021 | −1.91 | 0.056 |
| HL model statistics | χ2 = 0.12; p-value of χ2 test = 0.998; AIC = 75.804; BIC = 86.144 | | | |
| LL model statistics | χ2 = 15.26; p-value of χ2 test = 0.004; AIC = 60.671; BIC = 71.011 | | | |

Table S16. Results of two separate logistic regressions testing low (LL) and high (HL) memory load trials independently. In these regressions, self-control success in a subsequent food choice task was the dependent variable. Pre-choice activities in NAc, VTA, and putamen were included as the regressors in both analyses.

***Exploration of memory load conditions on brain activity***

In this study, working memory conditions served as the tasks preceding food choices, which were helpful in studying the pre-choice brain activity (see Participants and Procedure). Consequently, the effects of this manipulation on pre-choice activity were not a focus of interest. It is noteworthy, however, that a higher level of pre-choice activity was observed in the VTA, left putamen, and right NAc in the high memory load condition (memorizing a 7-digit number) compared to the low memory load condition (memorizing a 1-digit number). These findings are in line with those of previous studies, which have demonstrated the involvement of midbrain and striatal dopaminergic activities in the processes of gating and updating the representations in working memory (Schultz, et al., 1993; D’Ardenne et al., 2012; Yu et al., 2013). A higher activity in our selected ROIs during more cognitively demanding compared to less demanding working memory task could be also explained by the study showing that dopaminergic activity during working memory task correlated with working memory load (Salami et al., 2019).

**References**

D’Ardenne, K., Eshel, N., Luka, J., Lenartowicz, A., Nystrom, L. E., & Cohen, J. D. (2012). Role of prefrontal cortex and the midbrain dopamine system in working memory updating. *Proceedings of the National Academy of Sciences*, 109(49), 19900-19909.

Schultz, W., Apicella, P., & Ljungberg, T. (1993). Responses of monkey dopamine neurons to reward and conditioned stimuli during successive steps of learning a delayed response task. *Journal of neuroscience*, 13(3), 900-913.

Yu, Y., FitzGerald, T. H., & Friston, K. J. (2013). Working memory and anticipatory set modulate midbrain and putamen activity. *Journal of Neuroscience*, 33(35), 14040-14047.
